# Maternal High-Fat Diet Impairs Placental Fatty Acid β-Oxidation and Metabolic Homeostasis in the Offspring

**DOI:** 10.3389/fnut.2022.849684

**Published:** 2022-04-14

**Authors:** Ling Zhang, Ziwei Wang, Honghua Wu, Ying Gao, Jia Zheng, Junqing Zhang

**Affiliations:** Department of Endocrinology, Peking University First Hospital, Beijing, China

**Keywords:** maternal high-fat diet, placenta, fatty acid β-oxidation, AMPK, offspring

## Abstract

Maternal overnutrition can affect fetal growth and development, thus increasing susceptibility to obesity and diabetes in later life of the offspring. Placenta is the central organ connecting the developing fetus with the maternal environment. It is indicated placental fatty acid metabolism plays an essential role in affecting the outcome of the pregnancy and fetus. However, the role of placental fatty acid β-oxidation (FAO) in maternal overnutrition affecting glucose metabolism in the offspring remains unclear. In this study, C57BL/6J female mice were fed with normal chow or high-fat diet before and during pregnancy and lactation. The placenta and fetal liver were collected at gestation day 18.5, and the offspring's liver was collected at weaning. FAO-related genes and AMP-activated protein kinase (AMPK) signaling pathway were examined both in the placenta and in the human JEG-3 trophoblast cells. FAO-related genes were further examined in the liver of the fetuses and in the offspring at weaning. We found that dams fed with high-fat diet showed higher fasting blood glucose, impaired glucose tolerance at gestation day 14.5 and higher serum total cholesterol (T-CHO) at gestation day 18.5. The placental weight and lipid deposition were significantly increased in maternal high-fat diet group. At weaning, the offspring mice of high-fat diet group exhibited higher body weight, impaired glucose tolerance, insulin resistance and increased serum T-CHO, compared with control group. We further found that maternal high-fat diet downregulated mRNA and protein expressions of carnitine palmitoyltransferase 2 (CPT2), a key enzyme in FAO, by suppressing the AMPK/Sirt1/PGC1α signaling pathway in the placenta. In JEG-3 cells, protein expressions of CPT2 and CPT1b were both downregulated by suppressing the AMPK/Sirt1/PGC1α signaling pathway under glucolipotoxic condition, but were later restored by the AMPK agonist 5-aminoimidazole-4-carboxyamide ribonucleoside (AICAR). However, there was no difference in CPT2 and CPT1 gene expression in the liver of fetuses and offspring at weaning age. In conclusion, maternal high-fat diet can impair gene expression involved in FAO in the placenta by downregulating the AMPK signaling pathway, and can cause glucose and lipid dysfunction of offspring at weaning, indicating that placental FAO may play a crucial role in regulating maternal overnutrition and metabolic health in the offspring.

## Introduction

Diabetes and obesity are worsening problems worldwide, and the onset of metabolic diseases could be associated with an abnormal development environment in early life (1–3). In recent years, unhealthy diets, maternal diabetes, obesity, and excess gestation weight gain have been identified as the common and preventable risk factors that determine susceptibility to obesity and diabetes in the offspring (2, 3). Maternal nutrition during pregnancy and lactation is correlated with fetal and neonatal growth (4–7). Our previous study found that maternal overnutrition was associated with glucose intolerance, insulin intolerance, hyperglycemia and hyperlipidemia in the offspring (6–9). Increasing studies also indicate that this transgenerational effect even can be transmitted to the next generation (10–12). However, the underlying mechanisms remain largely unknown.

Placenta is a transient support organ that controls the crosstalk between mother and offspring (13, 14). Changes in placental function can alter the supply of key nutrients hormones, reactive oxygen species and inflammatory cytokines to the fetus (13, 15–17). It is indicated placental fatty acid metabolism plays an essential role in affecting the outcome of the pregnancy and fetus. Studies have presented that decreased fatty acid β-oxidation (FAO) in the placenta can lead to intracellular lipid accumulation, affecting fetal fatty acid delivery and altering fetal growth and development (18, 19). The process of FAO consists of two steps (20–22). At the first step, fatty acids are activated into acyl-coenzyme A (acyl-CoA) esters in the cytoplasm and then transported to the mitochondrial matrix. This step is determined by the carnitine palmitoyltransferase (CPT) system. The expression and activity of CPT1 and CPT2 are key factors that can regulate FAO. CPT1 is an enzyme located in the outer membrane of mitochondria which transfers the acyl group from acyl-CoA to carnitine (21). CPT2 is an enzyme located in the inner membrane of mitochondria which recovers acylcarnitine into acyl-CoA esters (21). At the second step, acyl-CoA is hydrolyzed into acetyl-CoA controlled by a series of enzymes, such as long chain acyl-CoA dehydrogenase (LCAD) and long-chain 3-hydroxyacyl-coa dehydrogenase (LCHAD). Then, acetyl-CoA enters the tricarboxylic acid (TCA) cycle to be completely oxidized to yield adenosine triphosphate (ATP). Several factors can influence the development of FAO in different tissues (23), of which AMP-activated protein kinase (AMPK) plays an essential role in regulating FAO (20). AMPK can enhance sirtuin 1 (Sirt1) activity, resulting in the deacetylation and modulation of the activity of peroxisome proliferator-activated receptor gamma coactivator 1-alpha (PGC1α) (24). Then, PGC1α can modulate the expression of CPT1 and CPT2 by recruiting peroxisome proliferator-activated receptors (PPARs) (24–27), such as peroxisome proliferator-activated receptor γ (PPAR γ) (27).

It is reported that FAO capacity is decreased in the placenta from women with obese and gestational diabetes mellitus (28–31). However, the effects and underlying mechanisms of maternal overnutrition on placental FAO and metabolic health in the offspring have not been fully elucidated. And little is known whether maternal overnutrition affects the FAO capacity of the offspring at fetal and weaning stage. Thus, our purpose is to investigate the impact of maternal overnutrition on placental gene expression involved in FAO and the role of the AMPK/Sirt1/PGC1α signaling pathway in regulating gene expression involved in FAO, and to further explore whether maternal overnutrition can affect hepatic FAO gene expression at the fetal and weaning stage in the offspring.

## Materials and Methods

### Ethics Statement

All experimental procedures were carried out in compliance with the Ethics Committee for Animal Experimentation of the Faculty of Peking University First Hospital (NO. J201827).

### Animals and Diets

Five-week-old female C57BL/6J mice were raised under specific pathogen free (SPF) conditions (12 h light-dark cycle; 22 ± 2°C). All animals had unlimited access to water and food. After 1 week of acclimation, all female mice were randomly assigned either to a normal chow (NC) diet group or to a high-fat (HF) diet group. The NC diet contained (kcal %): fat, 13%; protein, 24%; carbohydrate, 63%. and delivered 3.44 kcal/g of energy (Keao Xieli Feed Co. Ltd., Beijing, China, Supplementary Table 1). The HF diet contained (kcal %): fat, 60%; protein, 20%; carbohydrate, 20%. and delivered 5.24 kcal/g of energy (Keao Xieli Feed Co. Ltd, Beijing, China, Supplementary Table 1). After 4 weeks of feeding, mating was performed by housing female mice with male mice for 4 days with a NC diet (female: male = 2:1), in order to eliminating the confounding effects of sires' diets. Vaginal plugs were checked every morning, and the presence of vaginal plugs was considered as day 0.5 of pregnancy. The pregnant mice (F0) were raised individually and remained their respective diets throughout gestation. There were two cohorts of dams in this study. In the first cohort, dams were anesthetized with pentobarbital and euthanized after 10 h of fasting on day 18.5 of pregnancy. Dams' blood samples were collected from the intraorbital retrobulbar plexus for further serum biochemical analysis. The placenta and fetuses were quickly dissected and weighed. Placenta and livers of fetuses were stored at −80°C. In the second cohort, the pregnant females remained their respective diets throughout gestation and lactation. The number of pups in each litter is 6–10. To avoid nutritional bias between litters, all litters were culled to six pups on day 1 after birth. On day 21, all pups were weaned. Body weight and fasting blood glucose (FBG) of offspring were examined weekly from birth to weaning. To prevent confounding causes associated the estrus cycle and hormone profile of female offspring, we paid close attention to male offspring in the present study. On day 21, one male offspring from every litter was selected for further analysis. After 10 h of fasting, the offspring were anesthetized with pentobarbital and euthanized. Blood samples were collected from the intraorbital retrobulbar plexus in anesthetized mice. Livers were collected and stored at −80°C for future analysis.

### Glucose Tolerance Tests in Dams and Offspring Mice

Oral glucose tolerances tests (OGTTs) were carried out in dams after 6 h of fasting, in order to reduce stress for dams. Intraperitoneal glucose tolerance tests (IPGTTs) were performed in male offspring after 10 h of fasting at weaning age. Before giving a glucose administration, blood glucose levels were monitored in the tail vein using a glucometer and glucose test strips (Contour TS, Bayer, Beijing, China) as the baseline level (time 0). Then blood glucose levels were monitored at 15, 30, 60, 120 min after given a glucose administration (2 g/kg body weight). The area under the curve (AUC) of OGTTs and IPGTTs was calculated as previously described (6).

### Serum Biochemical Parameters Measurement

Blood samples taken from dams and male offspring were centrifuged at 4,000 g for 15 min and stored at −80°C. Serum samples were subjected to detect insulin concentrations using the Mouse Ultrasensitive Insulin ELISA kit (80-INSMSU-E01, ALPCO Diagnostics, Salem, NH, USA). Serum total cholesterol (T-CHO), triacylglycerol (TG), free fatty acids (FFA) were detected using commercial kits (A111-1, A110-1, A402-2-1, Jiancheng Bioengineering Institute, Nanjing, China). Each sample was detected in duplicate.

### Oil Red O Staining

The frozen placenta was embedded in Tissue-Tek O.C.T.Compound. Samples were cut at a thickness of 10 μm from each sample. Slides were washed in distilled water for 2 min and 60% isopropanol for 2 min. Afterward, slides were stained for 10 min in 60% working Oil Red O (G1015, ServiceBio, Beijing, China). Then slides were washed shortly with distilled water and 60% isopropanol, stained for 1 min with Hematoxylin (C0107, Beyotime, Shanghai, China), washed in running water for 10 min, mounted using glycerol jelly mounting medium (C0187, Beyotime, Shanghai, China). Images were captured using an Olympus DP71 microscope. Five random fields of view per tissue section were used to quantify the integrated optical density (IOD) using ImageJ software.

### Histological Analysis

Placental tissues embedded in wax were sliced at a thickness of 5 μm. For the hematoxylin and eosin (H&E) assay, the slides were stained with hematoxylin for 5 min and eosin Y for 30 s (C0105S, Beyotime, Beijing, China). Images were captured using an Olympus DP71 microscope. For immunohistochemistry assay, antigen retrieval was carried out in citrate buffer pH 6.0 (P0083, Beyotime, Shanghai, China) using a pressure cooker for 3 min, followed by blocking endogenous peroxidases using 0.3% H_2_O_2_ for 15 min. After blocking 20 min by blocking buffer (P0260, Beyotime, Shanghai, China), the slides were incubated overnight at 4°C with an anti-CPT2 antibody (1:150, ab181114, Abcam, Cambridge, UK), anti-CPT1b antibody (1:150, 22170-1-AP, Proteintech, Wuhan, China). On the next day, the slides were washed and then incubated with a secondary antibody (PV9001, Zhongshan Gold Bridge Biotechnology Co, Beijing, China) for 20 min at room temperature. The slides were incubated with DAB (ZLI9018, Zhongshan Gold Bridge Biotechnology Co, Beijing, China) to detect side-specific antigen-antibody binding, followed by staining with hematoxylin. Then, the slides were dehydrated and sealed with neutral gum. Images were captured using an Olympus DP71 microscope. Five random views per tissue section were used to quantify the mean IOD (IOD/area) using Image-Pro Plus 6.0.

### RNA Preparation and RT-PCR Analysis

Total RNA from placenta tissues and liver tissues was extracted using TRIzol reagent (15596026, Invitrogen, Waltham, MA, USA) and 1.5 μg of RNA was reversed to cDNA using the High-Capacity cDNA Reverse Transcription Kits (4375222, ThermoFisher Scientific Hudson, NH, USA). Real-time PCR was carried out using 15 ng of cDNA to detect the gene expression of CPT2, CPT1a, CPT1b, LCAD, LCHAD, AMPKα, Sirt1, PGC1α, PPARγ and mitochondrial transcription factor A (TFAM). β-actin was selected as the reference gene. Primers were presented in Table 1. The 2^−ΔΔCt^ method was used to calculate gene relative expression.

**Table 1:** Primer sequences of study genes.

**Genes**	**Forward**	**Reverse**
AMPKα	GTCAAAGCCGACCCAATGATA	CGTACACGCAAATAATAGGGGTT
Sirt1	ATGACGCTGTGGCAGATTGTT	CCGCAAGGCGAGCATAGAT
PGC1α	TATGGAGTGACATAGAGTGTGCT	CCACTTCAATCCACCCAGAAAG
PPARγ	TCGCTGATGCACTGCCTATG	GAGAGGTCCACAGAGCTGATT
CPT1a	CTCCGCCTGAGCCATGAAG	CACCAGTGATGATGCCATTCT
CPT1b	GCACACCAGGCAGTAGCTTT	CAGGAGTTGATTCCAGACAGGTA
CPT2	CAGCACAGCATCGTACCCA	TCCCAATGCCGTTCTCAAAAT
LCHAD	TGCATTTGCCGCAGCTTTAC	GTTGGCCCAGATTTCGTTCA
LCAD	TCTTTTCCTCGGAGCATGACA	GACCTCTCTACTCACTTCTCCAG
TFAM	ATTCCGAAGTGTTTTTCCAGCA	TCTGAAAGTTTTGCATCTGGGT
β-actin	TATTGGCAACGAGCGGTTCC	GGCATAGAGGTCTTTACGGATGTC

*AMPKα, AMP-activated protein kinase α; Sirt1, sirtuin 1; PGC1α, peroxisome proliferator-activated receptor gamma coactivator 1-alpha; PPARγ, Peroxisome proliferator-activated receptor γ; CPT1a, carnitine palmitoyltransferase 1a; CPT1b, carnitine palmitoyltransferase 1b; CPT2, carnitine palmitoyltransferase 2; LCAHD, long-chain 3-hydroxyacyl-coa dehydrogenase; LCAD, long chain acyl-CoA dehydrogenase; TFAM, mitochondrial transcription factor A*.

### Protein Isolation and Western Blot Analysis

Total protein from placenta tissues was ground and lysed in RIPA lysis buffer (P0013, Beyotime, Shanghai, China) and recovered by centrifuging at 12,000 g for 15 min at 4°C. After quantifying the protein concentration using bicinchoninic acid (BCA) protein quantification kit (23225, Thermo Fisher Scientific, Hudson, NH, USA), 20 μg protein was run on a 10% acrylamide SDS-PAGE gel. Then the protein was transferred onto a PVDF or nitrocellulose membrane. After blocking with 5% milk, the membranes were incubated overnight at 4°C with anti-p-AMPKα (1:1000; #2535, Cell Signaling Technology, Danvers, MA, USA), anti-AMPKα (1:1000; #5831s, Cell Signaling Technology, Danvers, MA, USA), anti-Sirt1 (1:1000; #3931, Cell Signaling Technology, Danvers, MA, USA), anti-PGC1α (1:1000; #2178, Cell Signaling Technology, Danvers, MA, USA), anti-CPT2 (1:1000; ab181114, Abcam, Cambridge, UK), anti-CPT1b (1:1000; 22170-1-AP, Proteintech, Wuhan, China), and anti-PPARγ (1:1000; #2435, Cell Signaling Technology, Danvers, MA, USA). On the next day, membranes were incubated with a secondary antibody (1:5000; Zhongshan Golden Bridge Biotechnology Co, Beijing, China) for 1 h at room temperature. The images of bands were visualized and taken using an enhanced chemiluminescent (ECL) detection kit. β-actin (1:10,000, AC026, ABclonal, China) was used as the control for total protein. Densitometry analysis was performed using Image J software.

### Cell Culture and Glucolipotoxic Treatment

Human JEG-3 trophoblast cells were obtained from the Chinese National Infrastructure of Cell Line Resource and maintained in Eagle's minimum essential medium (EMEM) (No.30-3003, ATCC, Manassas, VA, USA). The medium was supplemented with 10% fetal bovine serum (FBS, Gibco, Life Science, Pittsburgh, PA, USA) and 1% penicillin-streptomycin (Sigma, Steinheim, Germany) at 37°C under 5% CO2.

Glucolipotoxicity treatment was a combination of 33.3 mM glucose, 400 μM oleic acid (OA, O7501, Sigma, Steinheim, Germany) and 400 μM linoleic acid (LA, L8134, Sigma, Steinheim, Germany) in the medium. Cells were seeded into 6-well plates at a density of 3 × 10^5^ cells/well. After attachment, cells were incubated for 24 h in either BSA or glucose/FFA or glucose/FFA/AMPK agonist 5-aminoimidazole-4-carboxamide ribonucleotide (AICAR) (0.5 mM, S1802, Selleckchem, USA) in the medium. The concentration and incubation period used in the experiment is based on previous studies (32–34). After treatment, cells were harvested in RIPA buffer and recovered by centrifuging at 12,000 g for 15 min. The protein sample was collected and quantified by a BCA protein quantification kit. Protein expression was detected using western blot as aforementioned.

### Statistical Analysis

GraphPad Prism 9.0 software was applied to calculate the data. Quantitative data were presented as the mean ± standard errors (SEM) for normally distributed variables. The difference between the two groups was performed with two-tailed Student's *t*-test. The difference among the three groups was performed with a one-way analysis of variance (ANOVA). OGTTs and IPGTTs were performed with a two-way ANOVA followed by Bonferroni's *post-hoc* test. A *P*-value <0.05 was considered statistically significant.

## Results

### Maternal HF Feeding Impaired Glucose and Lipid Metabolism in Dams

**Figure 1:**
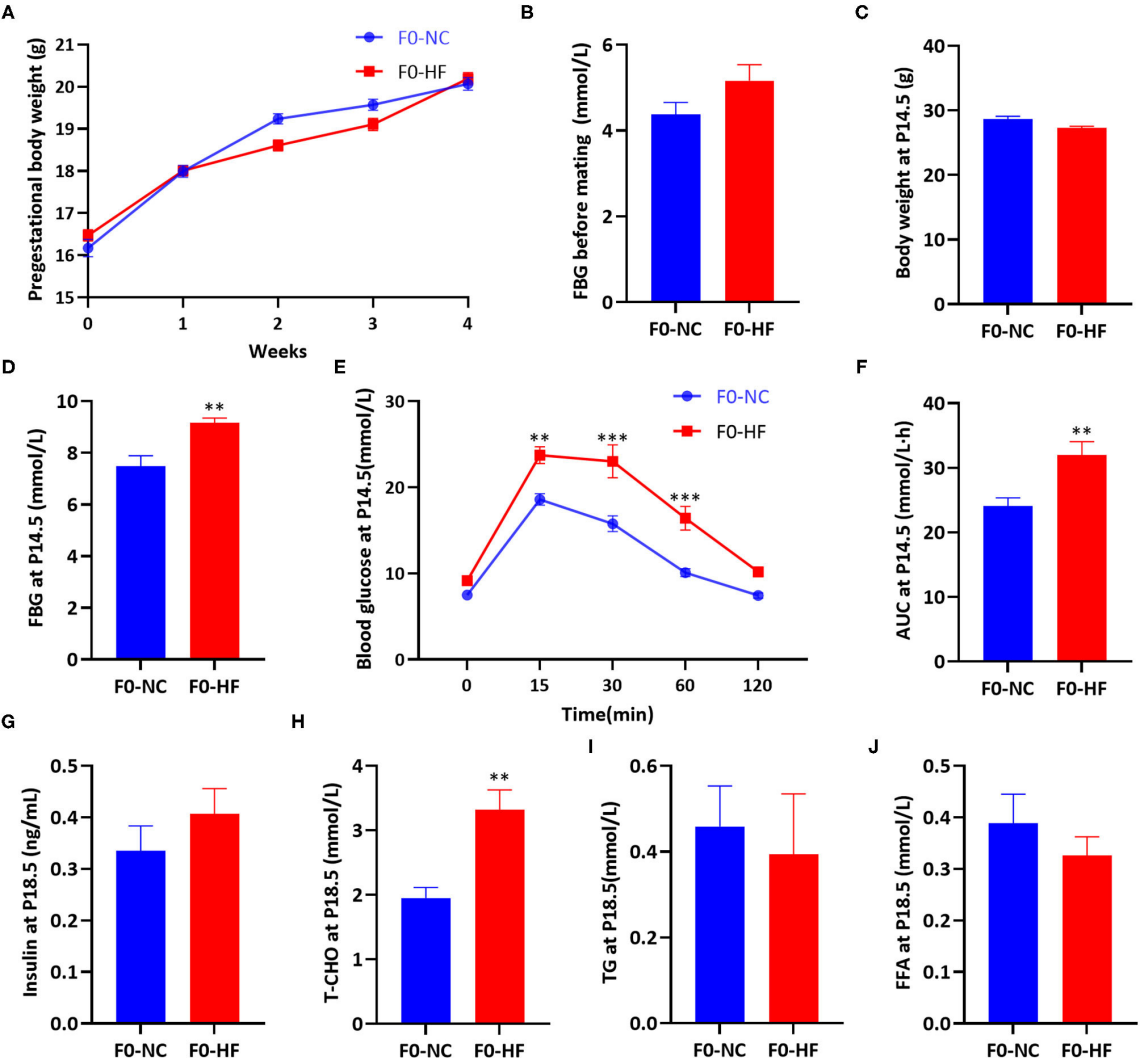
Maternal HF feeding impaired glucose and lipid metabolism in dams. **(A)** Body weight of dams during 4 weeks before mating; **(B**) FBG of dams before mating; **(C)** Body weight of dams at P14.5; **(D)** FBG of dams at P14.5; **(E)** OGTT of dams at P14.5; **(F)** AUC of dams at P14.5; **(G)** Serum insulin of dams at P18.5; **(H)** Serum T-CHO of dams at P18.5; **(I)** Serum TG of dams at P18.5; **(J)** Serum FFA of dams at P18.5. Data represented as the mean ± SEM. (F0-NC, *n* = 5–6; F0-HF, *n* = 6–7). *p < 0.05, **p < 0.01, ***p < 0.001 vs. F0-NC. NC, normal chow diet; HF, high-fat diet; FBG, fasting blood glucose; OGTT, oral glucose tolerances tests; AUC, the area under the glucose curve; T-CHO, total cholesterol; TG, triglyceride; FFA, free fatty acid.

Body weight and FBG before mating were not affected by maternal HF diet (*P* > 0.05, Figures 1A,B). At gestation day 14.5 (P14.5), no difference in body weight was observed between the two groups (*P* > 0.05, Figure 1C). However, HF-fed dams had higher FBG (*P* < 0.01, Figure 1D) and impaired glucose tolerance at P14.5. After oral glucose administration, HF-fed dams showed significantly higher glucose levels at 15 min (*P* < 0.01), 30 min (*P* < 0.001), and 60 min (*P* < 0.001) (Figure 1E). The overall glucose AUC was higher in HF dams compared with control group (*P* < 0.01, Figure 1F).

At gestation day 18.5 (P18.5), no significant difference in serum insulin levels of dams was observed between the two groups (*P* > 0.05, Figure 1G). Dams fed with HF diet had significantly increased serum T-CHO concentration (*P* < 0.01, Figure 1H). However, serum TG and FFA levels were similar between the two groups (*P* > 0.05, Figures 1I,J). These results indicated that maternal HF feeding before and during pregnancy could impair both glucose and lipid metabolism in dams.

### Maternal HF Feeding Altered the Development of Placenta and Fetus, and Induced Lipid Deposition in the Placenta

**Figure 2:**
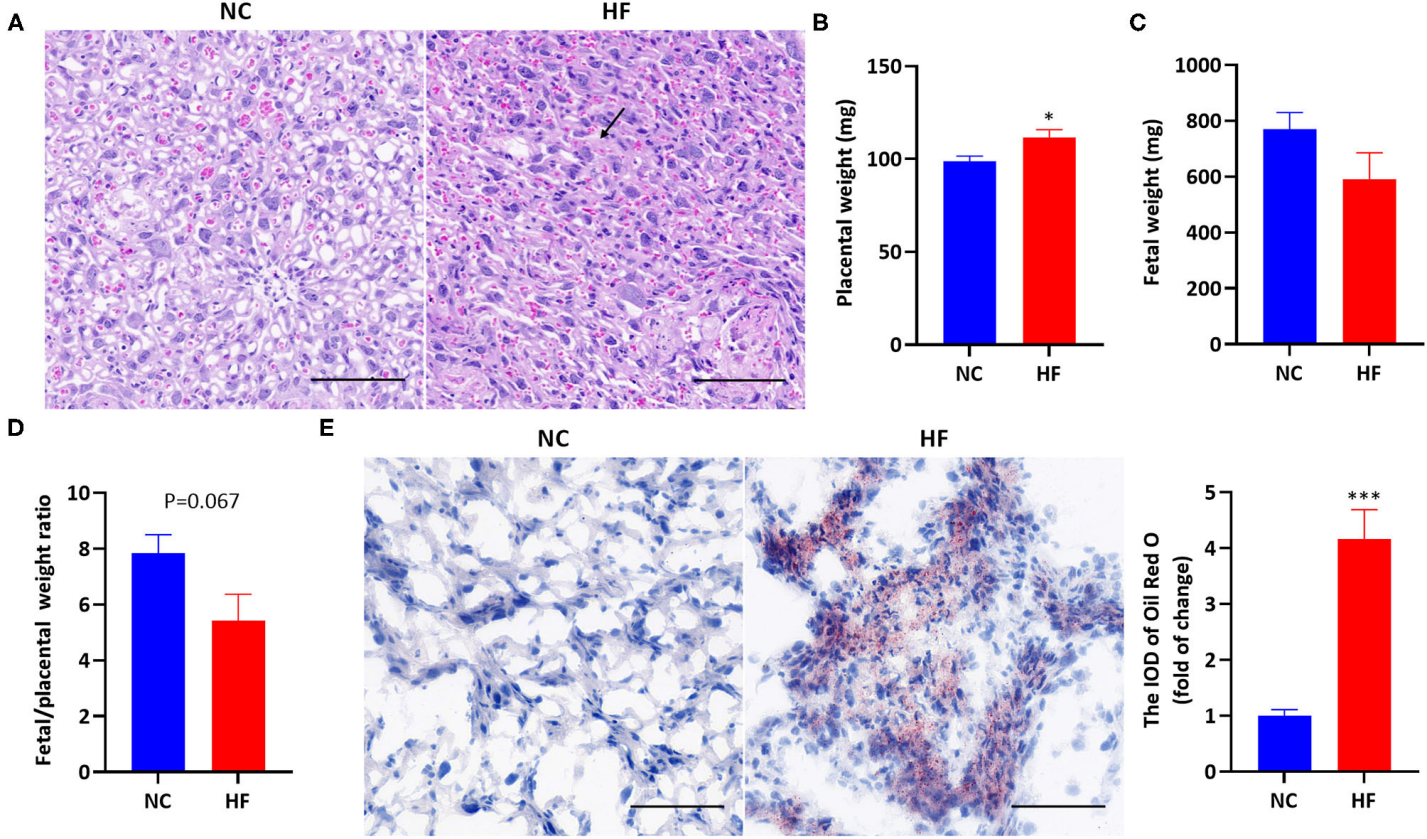
Maternal HF feeding altered the development of the placenta and fetal, and induced lipid deposition in the placenta. **(A)** Representative images of H&E staining in the placenta. The black arrow indicates interstitial edema, magnification 400x. The scale bar indicates 100 μm; **(B)** Placental weight; **(C)** Fetal weight; **(D)** Fetal/ placental weight ratio; **(E)** Representative images and quantitative assessment of Oil Red O staining in the placenta, magnification 400x. The scale bar indicates 100 μm. Data represented as the mean ± SEM. (NC, *n* = 6; HF, *n* = 6–7). *p < 0.05, ***p < 0.001 vs. NC. NC, normal chow diet; HF, high-fat diet; H&E, hematoxylin and eosin; IOD, integrated optical density.

Analysis of placental H&E staining revealed interstitial edema, enlarged blood sinusoid and architectural distortion in the labyrinth of the placenta in the HF feeding dams (Figure 2A). The average weight of each litter was used for the calculation of placental and fetal weight. The placental weight was significantly higher due to maternal HF diet (*P* < 0.05, Figure 2B). Fetal weight was not affected by maternal HF diet (*P* > 0.05, Figure 2C). However, there is a trend of decreased fetal/placental weight ratio in the HF diet group at P18.5 (*P* = 0.067, Figure 2D). For Oil Red O staining, HF dams presented a massive accumulation of large lipid droplets in the placenta, compared with the NC diet group (*P* < 0.001, Figure 2E).

### Maternal HF Feeding Impairs FAO by Suppressing AMPK Signaling Pathway in the Placenta

**Figure 3:**
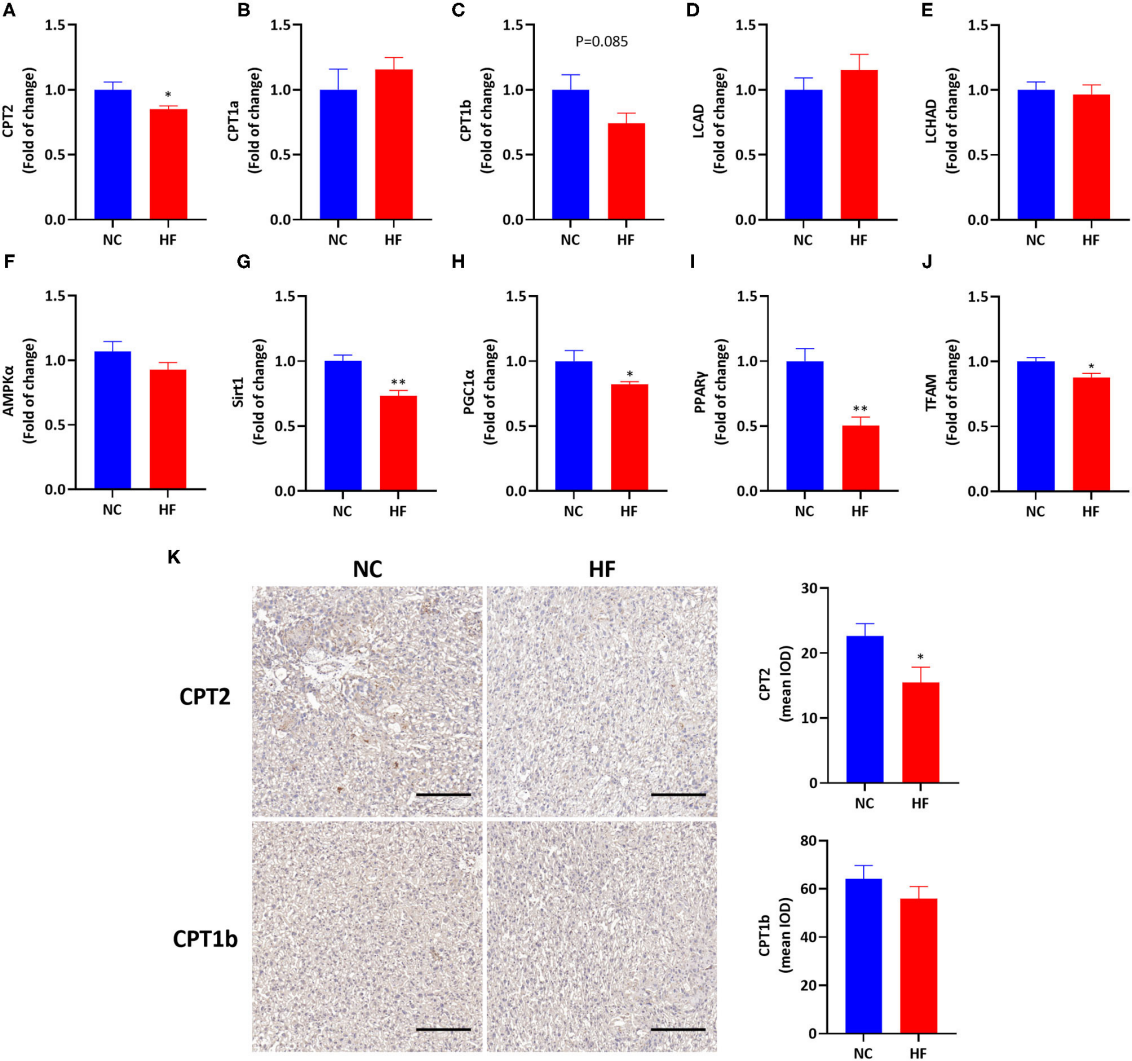
HF feeding reduced placental FAO gene expression by suppressing the AMPK signaling pathway. **(A–J)** Placental mRNA expression of genes. **(A)** CPT2; **(B)** CPT1a; **(C)** CPT1b; **(D)** LCAD; **(E)** LCHAD; **(F)** AMPKα; **(G)** Sirt1; **(H)** PGC1α; **(I)** PPARγ; **(J)** TFAM; **(K)** Immunohistological analysis of CPT2 and CPT1b in the placenta, magnification 200x. The scale bar indicates 200 μm. Data represented as the mean ± SEM. (NC, *n* = 5–6; HF, *n* = 7). *p < 0.05, **p < 0.01, vs. NC. NC, normal chow diet; HF, high-fat diet; CPT2, carnitine palmitoyltransferase 2; CPT1a, carnitine palmitoyltransferase 1a; CPT1b, carnitine palmitoyltransferase 1b; LCAHD, long-chain 3-hydroxyacyl-coa dehydrogenase; LCAD, long chain acyl-CoA dehydrogenase; AMPKα, AMP-activated protein kinase α; Sirt1, sirtuin 1; PGC1α, peroxisome proliferator-activated receptor gamma coactivator 1-alpha; PPARγ, peroxisome proliferator-activated receptor γ; TFAM, mitochondrial transcription factor A; IOD, integrated optical density.

To evaluate the effects of HF feeding on placental FAO, we measured mRNA expressions of related genes involved in FAO in the placenta. A maternal HF diet downregulated the gene expression of CPT2 (*P* < 0.05, Figure 3A), and had a trend of decreased CPT1b mRNA expression in the placenta (*P* = 0.085, Figure 3C). However, mRNA expression of CPT1a, LCAD, and LCHAD were similar in the placenta between the two groups (*P* > 0.05, Figures 3B,D,E). Then we measured related gene expression in the AMPK signaling pathway and found that there was no difference in placental AMPKα gene expression between the two groups (*P* > 0.05, Figure 3F). The mRNA expressions of Sirt1 (*P* < 0.01), PGC1α (*P* < 0.05), and PPARγ (*P* < 0.01) in the placenta were significantly decreased in the HF group (Figures 3G–I). Since FAO occurs in the mitochondria (21), we evaluated placental mitochondrial biogenesis by measuring the gene expression of TFAM. TFAM is a DNA-binding protein that plays a central role in transcriptional activation, mitochondrial DNA (mtDNA) organization and FAO capacity (35). The result showed that the mRNA expression of TFAM was reduced by HF die (*P* < 0.05, Figure 3J).

Then we applied immunohistology to determine the expression of CPT2 and CPT1b in the placenta. It was shown that maternal HF diet downregulated placental CPT2 expression (*P* < 0.05, Figure 3K). There was no significant difference in placental CPT1b expression between the HF diet and NC diet groups (*P* > 0.05, Figure 3K).

**Figure 4:**
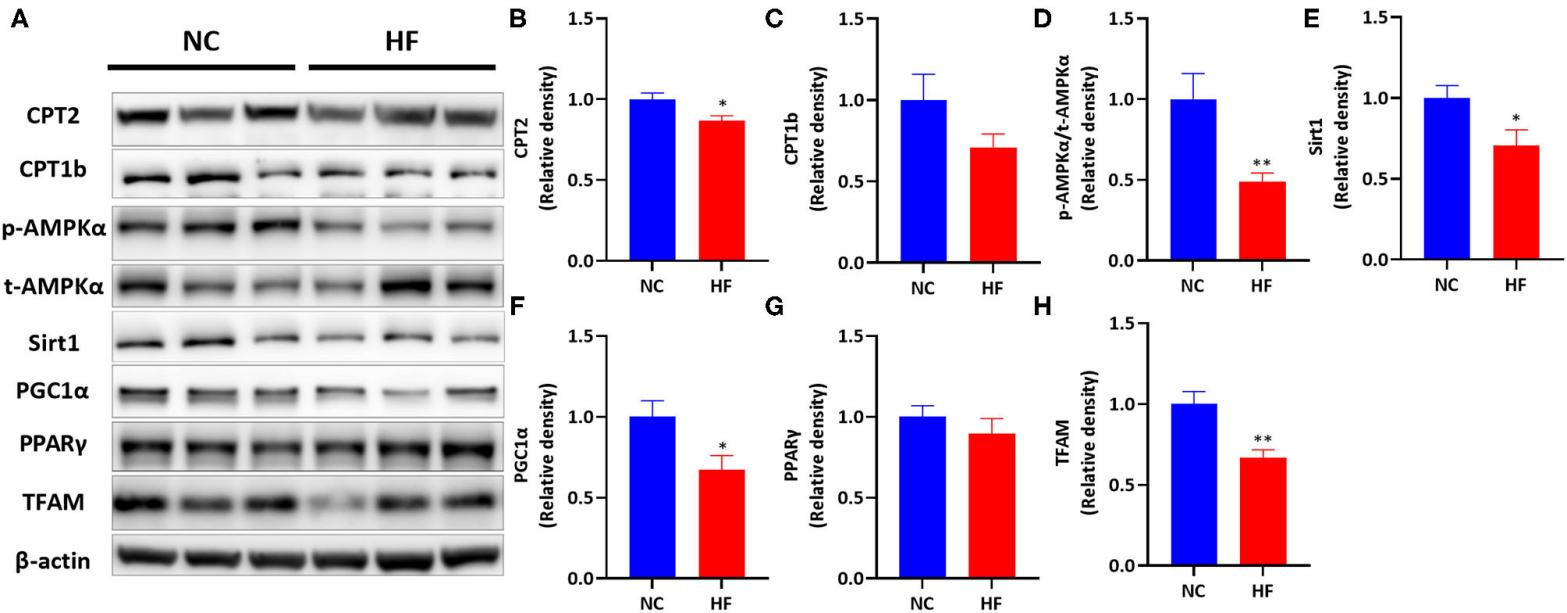
HF feeding reduced protein expression of placental FAO by suppressing the AMPK signaling pathway. **(A)** Bands' representative images of **(B–G)**. **(B)** CPT2; **(C)** CPT1b; **(D)** p-AMPKα/t-AMPKα; **(E)** Sirt1; **(F)** PGC1α; **(G)** PPARγ; **(H)** TFAM. Data represented as the mean ± SEM. (NC, *n* = 5–6; HF, *n* = 6–7). *p < 0.05, **p < 0.01, vs. NC. NC, normal chow diet; HF, high-fat diet; AMPKα, AMP-activated protein kinase α; Sirt1, sirtuin 1; PGC1α, peroxisome proliferator-activated receptor gamma coactivator 1-alpha; PPARγ, Peroxisome proliferator-activated receptor γ; CPT1a, carnitine palmitoyltransferase 1a; CPT1b, carnitine palmitoyltransferase 1b; CPT2, carnitine palmitoyltransferase 2; TFAM, mitochondrial transcription factor A.

Then we measured the protein levels of the above genes (Figure 4). Maternal HF diet downregulated placental CPT2 protein expression (*P* < 0.05, Figure 4B) and did not affect CPT1b protein level (*P* > 0.05, Figure 4C). Then we measured the protein expression of the AMPK signaling pathway. A maternal HF diet did not alter placental total AMPKα (t-AMPKα) protein level but downregulated placental phosphorylated AMPKα (p-AMPKα) level. The p-AMPKα/t-AMPKα ratio was significantly decreased in the placenta of maternal HF diet (*P* < 0.01, Figure 4D). Maternal HF diet downregulated placental Sirt1 (*P* < 0.05, Figure 4E), and PGC1α (*P* < 0.05, Figure 4F) protein expression. However, there was no difference in placental PPARγ protein level between the NC and HF groups (*P* > 0.05, Figure 4G). Meanwhile, the protein expression of TFAM in the placenta was decreased by HF diet (*P* < 0.05, Figure 4H).

### Glucolipotoxicity Reduced FAO by Suppressing AMPK Signaling Pathway and Restored by AICAR

**Figure 5:**
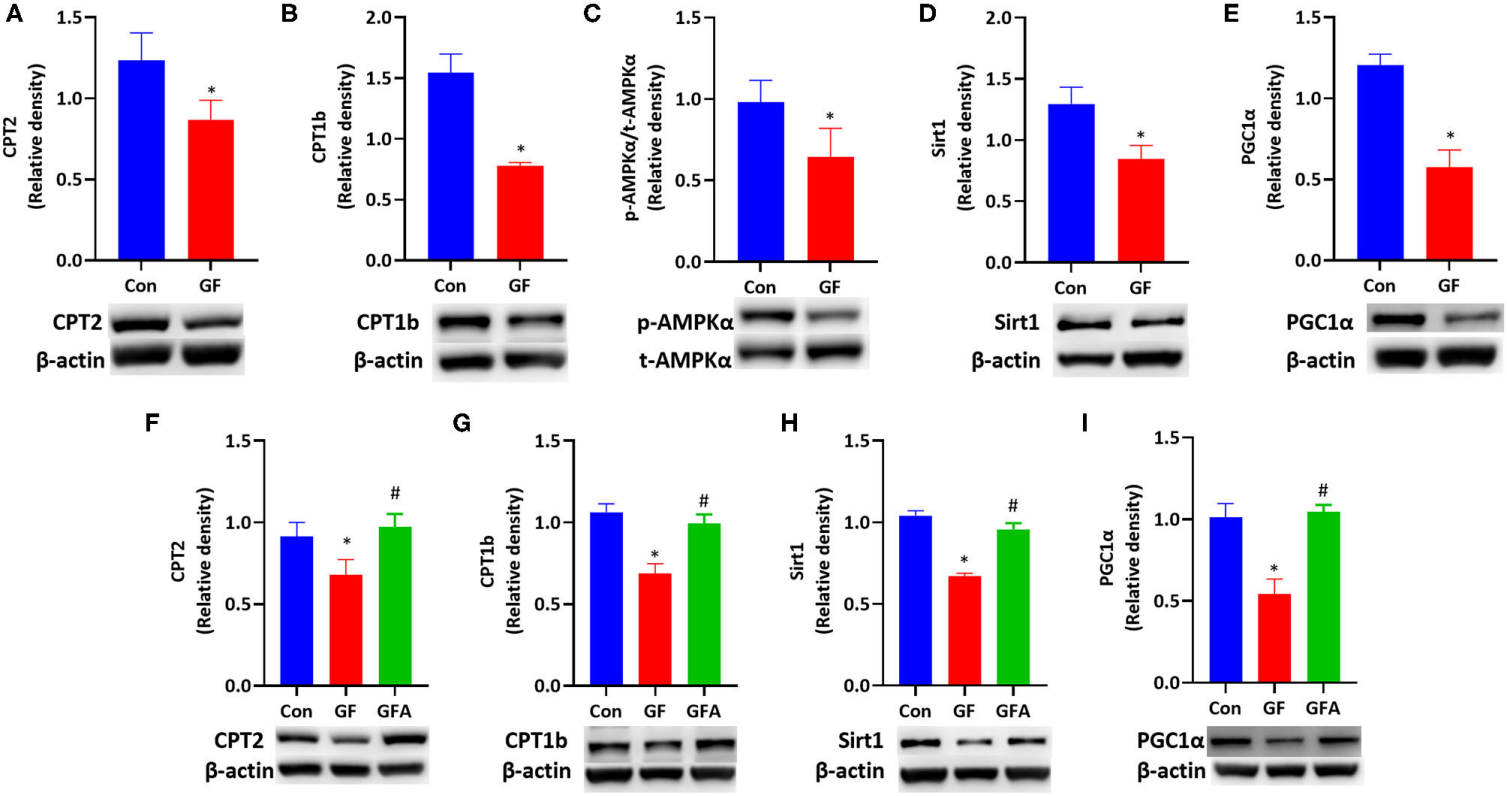
Glucolipotoxicity reduced FAO-related protein expression by suppressing the AMPK signaling pathway and reversed by AICAR. **(A–E)** JEG-3 cells were treated with BSA (Con) or glucose/FFA (GF), which were treated for 10 min for AMPKα and treated for 24h for other proteins. **(A)** CPT2; **(B)** CPT1b; **(C)** p-AMPKα/t-AMPKα protein ratio; **(D)** Sirt1; **(E)** PGC1α. **(F–I)** JEG-3 cells were treated with BSA (Con) or glucose/FFA (GF) or glucose/FFA/AICAR (GFA) for 24 h. **(F)** CPT2; **(G)** CPT1b; **(H)** Sirt1; **(I)** PGC1α. The experiment was repeated three-five times. Data represented as the mean ± SEM. *p < 0.05, vs. Con. ^#^*p* < 0.05, vs. GF. GF, glucose/FFA; GFA, glucolipotoxicity plus AICAR; AICAR, 5-aminoimidazole-4-carboxamide ribonucleotide; AMPKα, AMP-activated protein kinase α; Sirt1, sirtuin 1; PGC1α, peroxisome proliferator-activated receptor gamma coactivator 1-alpha; CPT1b, carnitine palmitoyltransferase 1b; CPT2, carnitine palmitoyltransferase 2.

As maternal HF feeding impaires glucose and lipid metabolism in mice, and hyperglycemia can enhance the toxicity of fatty acid termed glucolipotoxicity (36). We investigated the effect of glucolipotoxicity on FAO in trophoblasts. We treated JEG-3 cells with a combination of glucose and FFA mixture to induce glucolipotoxicity. During the 24 h incubation with glucolipotoxicity, glucolipotoxicity-treated cells responded with significantly downregulated CPT2 and CPT1b protein expression (*P* < 0.05, Figures 5A,B). Glucolipotoxicity did not alter t-AMPKα protein level. However, p-AMPKα levels were reduced by glucolipotoxicity in JEG-3 cells. The p-AMPKα/t-AMPKα ratio was decreased (*P* < 0.05, Figure 5C), which indicated that AMPK activity was suppressed by glucolipotoxicity. Then we evaluated the downstream of the AMPK signaling pathway, and found that glucolipotoxicity reduced the protein expressions of Sirt1 (*P* < 0.05, Figure 5D) and PGC1α (*P* < 0.05, Figure 5E) in JEG-3 cells.

To explore whether the effect of glucolipotoxicity on FAO is mediated by AMPK, we treated JEG-3 cells with AMPK activator, AICAR. The protein expressions of CPT2 (*P* < 0.05) and CPT1b (*P* < 0.05) were increased upon AICAR treatment (Figures 5F,G). Meanwhile, the suppressive effect in the protein expression of Sirt1 (*P* < 0.05) and PGC1α (*P* < 0.05) induced by glucolipotoxicity was also blocked by AICAR treatment (Figures 5H,I). These results suggested that glucolipotoxicity reduced FAO by suppressing the AMPK signaling pathway and restored by AICAR in JEG-3 cells.

### Maternal HF Feeding Impaired Glucose and Lipid Metabolism of Offspring at Weaning

**Figure 6:**
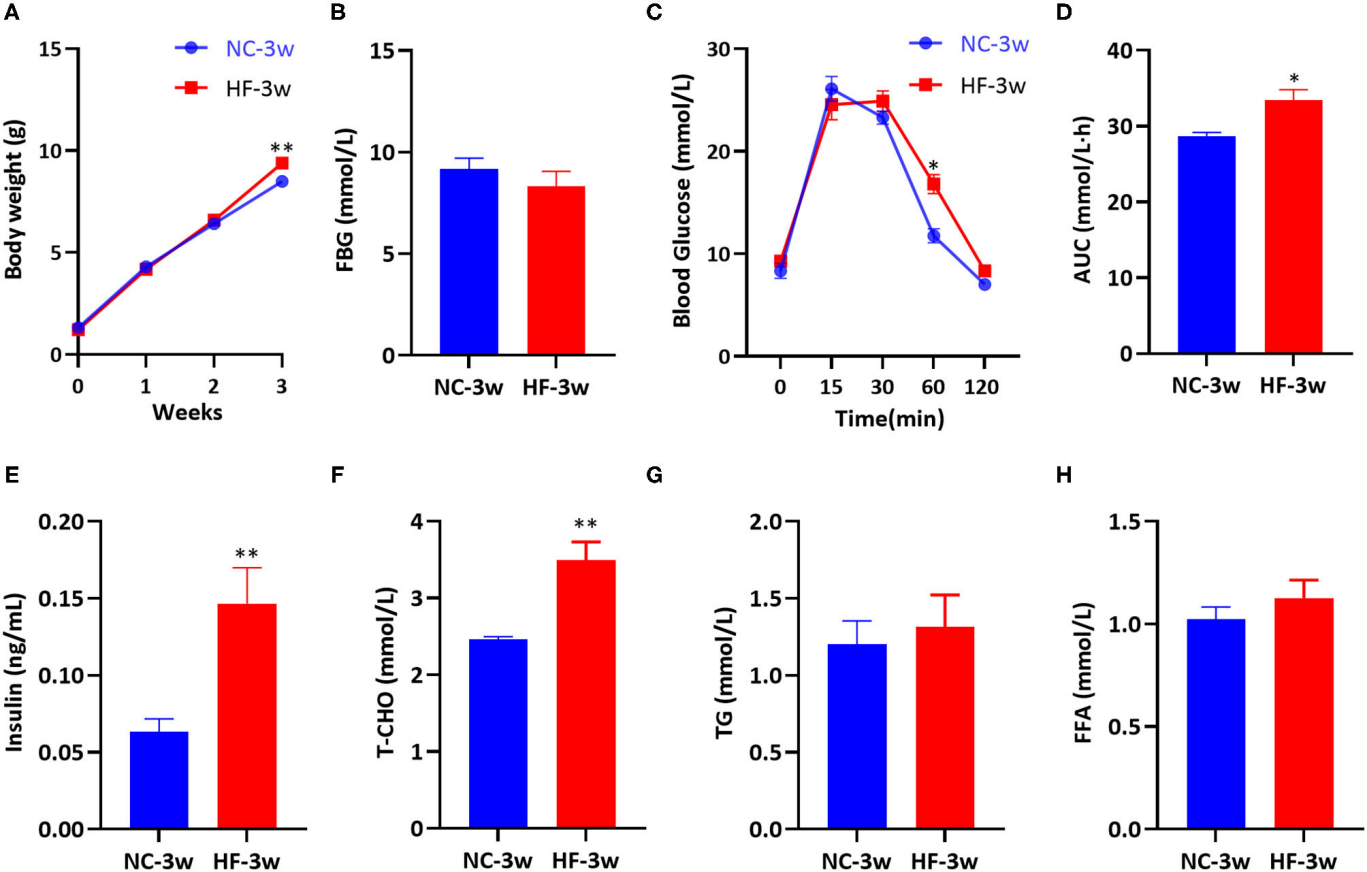
Maternal HF feeding impaired glucose and lipid metabolism of offspring at weaning. **(A)** Body weight of male offspring from birth to weaning; **(B)** FBG of male offspring at weaning; **(C)** IPGTT and **(D)** AUC of male offspring at weaning; **(E)** Serum insulin; **(F)** Serum T-CHO; **(G)** Serum TG; **(H)** Serum FFA of male offspring at weaning. Data represented as the mean ± SEM. (NC-3w, *n* = 8; HF-3w, *n* = 6–8). *p < 0.05, **p < 0.01 vs. NC-3w. NC, normal chow diet; HF, high-fat diet; 3w, the third week; FBG, fasting blood glucose; IPGTT, intraperitoneal glucose tolerance test; AUC, the area under the glucose curve; T-CHO, total cholesterol; TG, triglyceride; FFA, free fatty acid.

After birth, we measured the offspring's body weight every week. No significant differences in body weight at birth, the first and the second week were observed between the male offspring of two groups. However, at weaning, male offspring presented increased body weight due to maternal HF diet (*P* < 0.05, Figure 6A). There was no substantial difference in FBG between the offspring of the two groups at weaning (*P* > 0.05, Figure 6B). Compared with the offspring of dams fed a NC diet, offspring of HF feeding dams had impaired glucose tolerance at weaning, which presented higher blood glucose levels at 60 min (*P* < 0.05, Figure 6C). The overall glucose AUC was higher in male offspring of HF feeding dams (*P* < 0.05, Figure 6D). Serum insulin concentration (*P* < 0.01, Figure 6E) and T-CHO concentration (*P* < 0.01, Figure 6F) were significantly higher in the offspring of dams fed a HF diet at weaning. However, serum TG and FFA levels were similar between the offspring of the two groups (*P* > 0.05, Figures 6G,H).

### Maternal HF Feeding Did Not Affect FAO Gene Expression in Offspring at Fetal and Weaning Age

**Figure 7:**
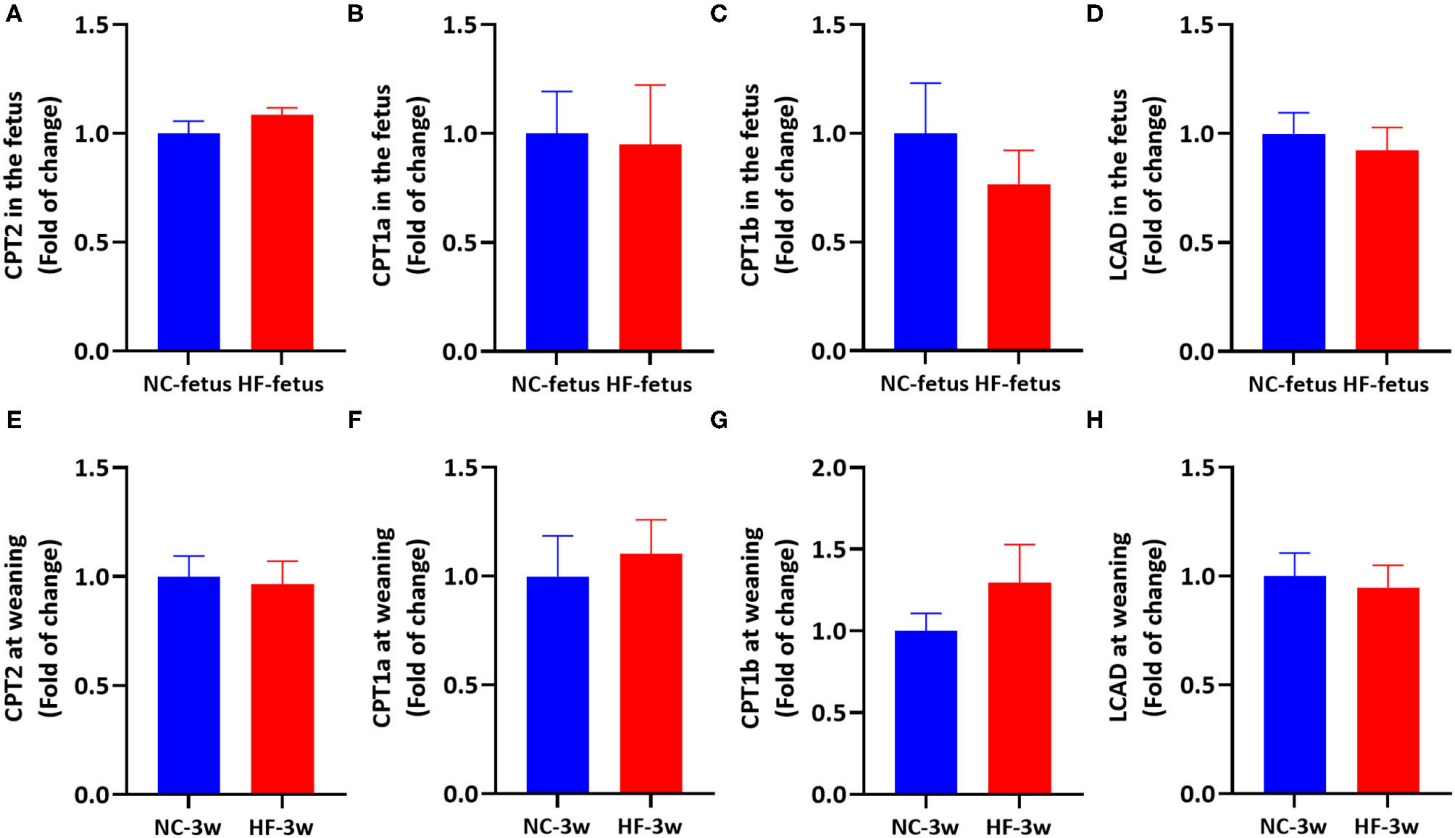
Maternal HF feeding did not affect FAO-related gene mRNA expression in offspring at fetal and weaning age. **(A–D)** hepatic mRNA expression in the fetus. **(A)** CPT2; **(B)** CPT1a; **(C)** CPT1b; **(D)** LCAD; **(E–H)** hepatic mRNA expression in the offspring at weaning. **(E)** CPT2, **(F)** CPT1a, **(G)** CPT1b, **(H)** LCAD. Data represented as the mean ± SEM. (NC-fetus, *n* = 6; HF-fetus, *n* = 7; NC-3w, *n* = 8; HF-3w, *n* = 8). NC, normal chow diet; HF, high-fat diet; 3w, the third week. CPT2, carnitine palmitoyltransferase 2; CPT1a, carnitine palmitoyltransferase 1a; CPT1b, carnitine palmitoyltransferase 1b; LCAD, long chain acyl-CoA dehydrogenase.

Apart from CPT1 and CPT2, carnitine is an important cofactor in transferring acyl-CoA from the cytoplasm to mitochondrial membranes in carnitine cycle system (21), which can stimulate the rate of transcription CPT1 and CPT2 (37), as well as the enzyme activity of CPT1 in the liver (37–39). Since the fetus and sucking infant have a very limited ability to synthesize carnitine, it is largely dependent on the transfer of carnitine from the placenta in the uterus (23) and breast milk after birth (40, 41). Previous studies showed that obese women exhibited lower placental total carnitine content (free carnitine and acylcarnitines) accompanied by defects of FAO, and reduced umbilical venous plasma total carnitine content (29). And it was reported that maternal HF feeding reduced the level of free-carnitine, short-chain and medium-chain acylcarnitines in the placenta (42), and decreased the level of L-carinitine in the liver of offspring (43). Oral L-carnitine administration can increase AMPK protein expression (44, 45). Thus, we supposed that maternal HF feeding may affect the FAO in the offspring through imbalanced carnitine. To test this XX, we measured the expression of hepatic genes related to FAO in the fetus and offspring at weaning. However, mRNA expression of CPT2, CPT1a, CPT1b, and LCAD in the liver were similar between the two groups' offspring at fetal and weaning age (*P* > 0.05, Figure 7). Then, we tested the AMPK signaling pathway in the liver to evaluate whether AMPK signaling pathway was activated. Correspondingly, there was no difference in hepatic AMPK, Sirt1 and PGC1α mRNA expression between the NC and HF groups (Supplementary Figure 1). These results indicated that AMPK/Sirt1/PGC1α pathway was not activated, so that FAO related gene expression was not altered.

## Discussion

Maternal overnutrition is common in developed countries and developing nations (46), which increases the risk of gestational diabetes mellitus and determines susceptibility to obesity and diabetes in the offspring (3). FAO defects can cause energy deficiency, accumulated substrates and oxidative stress (47). However, the effect and mechanism of maternal overnutrition on FAO in the placenta has not been fully depicted, and studied about whether FAO is altered in the liver of the offspring are limited, especially during fetal age and weaning age. The present study showed that maternal HF feeding could induce glucose and lipid metabolism disorders in dams. Compared with the NC diet group, a maternal HF diet caused heavier placenta and a decreased trend of fetal/placental weight ratio. In addition, a maternal HF diet decreased the gene expression involved in FAO by inhibiting the AMPK/Sirt1/PGC1α signaling pathway in the placenta. In addition, a maternal HF diet caused a higher body weight, glucose intolerance, hyperinsulinemia and hypercholesteremia of offspring at weaning age. However, FAO-related genes expression in the liver of fetal and offspring at weaning age had not been altered.

The placenta plays a central role in linking mother and fetus (13, 14). Development and metabolic requirement of the placenta and fetus can be changed to meet the pregnant mother's metabolism and nutrient availability (15, 48). Fetal growth is influenced by the process of placental nutrient transfer. Fetal/placental weight ratio can be an indicator of placental efficiency (49). The present study showed that the maternal HF diet group had a heavier placenta and a decreased trend of fetal/placental weight ratio, which indicated impaired placental efficiency in transport nutrition. In line with our study, several studies have reported that a pregestational and/or gestational exposure to the maternal HF diet resulted in increased placental weight and decreased fetal weight, which is characterized by growth restriction (6, 50, 51). Low birth weight is correlated with a higher risk of diabetes, obesity and other metabolic diseases in later life (7, 9, 52). Though birth weight was not affected by maternal HF feeding, the offspring did exhibit higher body weight, impaired glucose tolerance, hyperinsulinemia and hypercholesteremia at weaning age in the present study. These results suggest that maternal HF feeding has profound consequences for the offspring later in life.

Fatty acids act as metabolic fuel and an energy source in the placenta (53). A deficiency of mitochondrial FAO can result in the accumulation of upstream metabolites, impaired ATP production, increased reactive oxygen species levels and inflammatory cytokines, which might be transferred to the fetus (54, 55). Furthermore, a computational experiment explored the rate-determining processes of fatty acid transfer across the placenta in isolated perfused human placenta (19). They found that the rate of fatty acid delivery to the fetal compartment was modulated by the incorporation of fatty acids into placental lipid pools (19). FAO reduction can shift the flux of fatty acids away from oxidation toward lipid pools (30), which suggests that FAO capacity can affect fatty acid delivery to the fetus (29, 56). There are studies showed that FAO capacity was reduced in trophoblast cells isolated from obese women (29), and acylcarnitine concentration and CPT1 and/or CPT2 expression were lower in the placenta (28). Placenta explants from women with gestational diabetes mellitus exhibited a reduction in FAO capacity (30, 57). Placental explants exposed to high glucose levels showed impaired FAO capacity by phosphorylation acetyl-CoA carboxylase and increased triglyceride accumulation from normal pregnancy women (30). Corresponding with these results, our study demonstrated that the maternal HF diet group showed downregulated CPT2 expression and a decreased trend of CPT1b, which indicated impaired gene expression involved in FAO.

AMPK, which plays an essential role in energy metabolism, is a metabolic master regulator of FAO (58). High AMPK activity can enhance Sirt1 activity, resulting in the deacetylation and modulation of the activity of PGC1α (24). PGC1α is a transcriptional coactivator and can modulate the expression of many genes related to FAO, such as CPT1 and CPT2 (24–26, 59). We found a lower placental phosphorylated AMPKα ratio (p-AMPKα/ t-AMPKα) due to a maternal HF diet, which suppressed the expression of Sirt1 and PGC1α. To further investigate the molecular mechanism of whether gene expression involved in FAO was regulated by the AMPK signaling pathway, we used a glucose/FFA-induced glucolipotoxicity cell model. In the present study, we chose the JEG-3 cell line to study placental function. Extravillous trophoblasts, which connect maternal with fetal interfaces, are frequently used to investigate placental function (60). The results suggested that exposure to glucolipotoxicity downregulated protein expression of CPT1b and CPT2 through reduced AMPK activity and protein expression of Sirt1 and PGC1α. And the decreases in protein expression of Sirt1, PGC1α, CPT1b and CPT2 induced by glucolipotoxicity were significantly elevated by AICAR, indicating that impaired protein expression involved in FAO was restored by AICAR treatment.

Previous studies showed that hepatic FAO-related genes of offspring were reduced by maternal HF diet at the fetal (61) and weaning age (62). However, in our experiment, gene expression involved in FAO was not altered by maternal HF diet both in the fetus and the offspring at the weaning age. It is proposed that the duration of HF exposure, the ingredient of diets and maternal metabolic stage program the offspring differently (50, 63). These conflict results may be due to diet composition, duration of HF exposure and/or maternal metabolic state.

In conclusion, to the best of our knowledge, our study for the first time demonstrated that maternal high-fat diet can impair gene expression involved in FAO in the placenta by downregulating the AMPK signaling pathway and can cause glucose and lipid dysfunction of offspring at weaning. It is indicated that placental FAO may play a essential/crucial/central role in regulating maternal overnutrition and metabolic health in the offspring. These findings can advance our thinking about placental FAO capacity between maternal overnutrition and metabolic homeostasis in the offspring.

## Data Availability Statement

The datasets presented in this study can be found in online repositories. The names of the repository/repositories and accession number(s) can be found in the article/Supplementary Material.

## Ethics Statement

All experimental procedures were carried out in compliance with the Ethics Committee for Animal Experimentation of the Faculty of Peking University First Hospital (NO. J201827).

## Author Contributions

JZha and JZhe conceived and designed the experiments. JZhe, LZ, and ZW carried out the experiments. HW and YG analyzed the data. All authors were involved in writing the paper and had final approval of the submitted and published versions.

## Funding

This study was sponsored by National Natural Science Foundation of China (Nos. 81800703 and 81970701), Beijing Nova Program (No. Z201100006820117), Beijing Municipal Natural Science Foundation (No. 7184252 and No. 7214258), Peking University Medicine Seed Fund for Interdisciplinary Research, the Fundamental Research Funds for the Central Universities, China Diabetes Young Scientific Talent Research Project and Bethune-Merck Diabetes Research Fund of Bethune Charitable Foundation. The funders had no role in study design, data collection and analysis, or preparation of the manuscript.

## Conflict of Interest

The authors declare that the research was conducted in the absence of any commercial or financial relationships that could be construed as a potential conflict of interest.

## Publisher's Note

All claims expressed in this article are solely those of the authors and do not necessarily represent those of their affiliated organizations, or those of the publisher, the editors and the reviewers. Any product that may be evaluated in this article, or claim that may be made by its manufacturer, is not guaranteed or endorsed by the publisher.
