# Supplementary material for: Maternal High-Fat Diet Impairs Placental Fatty Acid β-Oxidation and Metabolic Homeostasis in the Offspring
Source: Front Nutr. 2022 Apr 14;9:849684. doi: 10.3389/fnut.2022.849684 (PMC9050107; doi:10.3389/fnut.2022.849684)
Supplement: Supplementary file 1 [file Data_Sheet_1.docx]

Supplementary Material

# Supplementary Figures and Tables

## Supplementary Table

Supplementary Table 1: Formulation of NC diet and HF diet.

| **Content** | **NC Diet (gm%)** | **HF Diet (gm%)** |
| --- | --- | --- |
| Carbohydrate | 48.26 | 25.04 |
| Protein | 22.53 | 26.23 |
| Fat | 5.10 | 34.89 |
| Fiber | 3.90 | 6.46 |
| Vitamin Mix | 0.764 | 1.55 |
| Mineral Mix | 4.72 | 5.82 |

gm%, gram percent

## Supplementary Figure

**
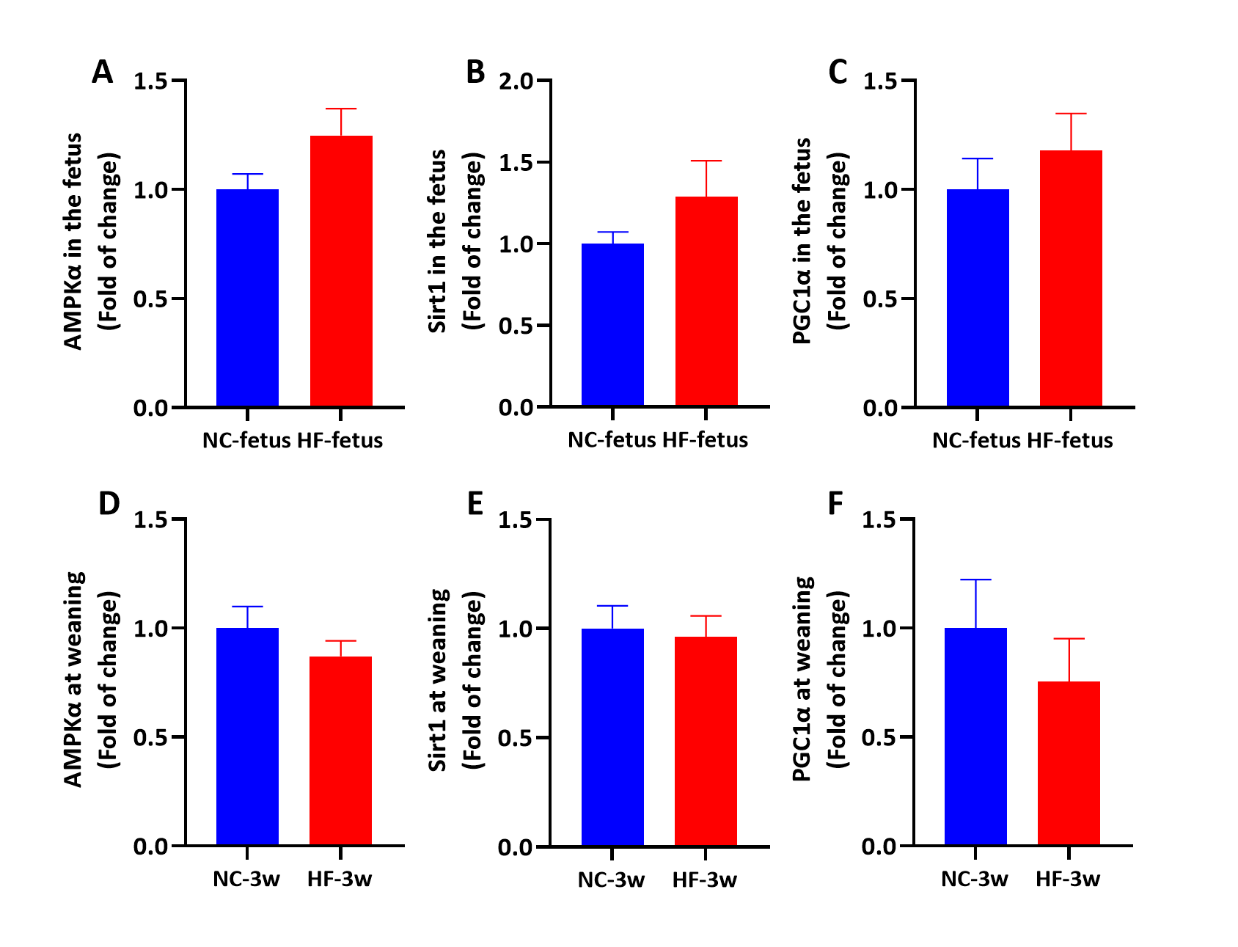
**

**Supplementary Figure 1.** **Maternal HF feeding did not affect hepatic mRNA expression of AMPK/Sirt1/PGC1α signaling pathway in offspring at fetal and weaning age.** **(A)** hepatic AMPKα mRNA expression in the fetus; **(B)** hepatic Sirt1 mRNA expression in the fetus; **(C)** hepatic PGC1αmRNA expression in the fetus; **(D)** hepatic AMPKα mRNA expression in the offspring at weaning; **(E)** hepatic Sirt1 mRNA expression in the offspring at weaning; **(F)** hepatic PGC1α mRNA expression in the offspring at weaning. Data represented as the mean ± SEM. (NC-fetus, n=6; HF-fetus, n=7; NC-3w, n=8; HF-3w, n=8). NC, normal chow diet; HF, high-fat diet; 3w, 3 weeks of age; AMPKα, AMP-activated protein kinase α; Sirt1, sirtuin 1; PGC1α, peroxisome proliferator-activated receptor gamma coactivator 1-alpha.
